# Embracing the Social Nature of Recovery: A Qualitative Study on the Resource Group Method for People With Severe Mental Illness

**DOI:** 10.3389/fpsyt.2020.574256

**Published:** 2020-11-24

**Authors:** Cathelijn D. Tjaden, Jenny Boumans, Cornelis L. Mulder, Hans Kroon

**Affiliations:** ^1^Department of Reintegration and Community Care, Trimbos Institute, Utrecht, Netherlands; ^2^Department of Social and Behavioral Sciences, Tranzo Scientific Center for Care and Welfare, Tilburg University, Tilburg, Netherlands; ^3^Department of Psychiatry, Erasmus Medical Center, Rotterdam, Netherlands; ^4^Antes, Parnassia Psychiatric Institute, Rotterdam, Netherlands

**Keywords:** recovery, social recovery processes, family involvement and experiences, empowerment, resource groups, severe mental illness (SMI), Assertive Community Treatment (A.C.T.), multiple case study approach

## Abstract

**Objective:** The resource group method for people with severe mental illness might provide a useful framework to facilitate patient's empowerment and systematically engage significant others. However, no research has explored the perspectives and experiences of patients and their significant others. This is crucial for better adjustment to the needs of the people using the method. The aim of this study was to develop a useful framework for a deeper understanding of the resource group method and its outcomes.

**Method:** The study used a longitudinal, qualitative multiple case-study design based on grounded theory methodology. During a period of 2 years, the developments and processes in eight resource groups were explored by conducting a total of 74 interviews (e.g., with patients, significant others, and mental health professionals) and 26 observations of resource group meetings.

**Results:** Analysis showed that a well-functioning resource group set the stage for five processes to unfold: (i) experience of support; (ii) acknowledgment of significant others; (iii) activation; (iv) openness; and (v) integration. These processes facilitated recovery both in terms of an arousing curiosity within the patient as well as increasing reciprocity and equality in their social relations. In addition, the method emphasized the uniqueness of each recovery journey, thereby providing a framework to shape recovery-oriented care. The analysis also revealed three hindering factors: (i) embedding and implementation issues; (ii) predominant network; and (iii) tensions inherent in the resource group setting.

**Conclusion:** Working according to the resource group method involves that the person's recovery work becomes a social process that takes place in relation to the social environment and everyday life in which it is important to acknowledge and integrate the needs of significant others in treatment and care. This study provides a first step toward a multidimensional comprehension of the resource group method, the working mechanisms and its influence on recovery for people with severe mental illness.

## Introduction

Flexible Assertive Community Treatment (FACT), a Dutch variant of Assertive Community Treatment [ACT; (1)], has been implemented throughout the Netherlands for patients with severe mental illness (SMI) experiencing problems in important domains in life (e.g., housing, finances, work, and social functioning). FACT is a service delivery model that combines highly intensive multidisciplinary treatment for clients at risk of relapse with moderate intensive care in times of stability (2). It has been argued that the current FACT teams can be enriched by integrating the Resource Group (RG) method into FACT for a more effective mobilization of patients' networks to achieve treatment and social inclusion goals (3). However, in-depth knowledge of the potential value of the RG method in the Dutch context of FACT is lacking.

According to the RG method, patients, significant others from their informal network (friends and family) and members of their formal network (social worker, nurse, case manager, psychiatrist, and peer worker) form an RG (4, 5). The RG meets quarterly to discuss the patients' recovery goals and wishes, and to jointly develop a plan to achieve them. The RG method is built around (re)capturing the patient's agency. Therefore, patients are encouraged to nominate those who will be included in the RG, define the recovery goals that determine the agenda of the meetings, and make decisions on how the meetings are designed (6). An important characteristic of the RG method is that significant others are systematically engaged in treatment and care (7). The treatment team no longer solely comprises care professionals but is augmented by the patient themselves, family members, friends, or others who are important to the patient. Mutual partnerships are developed and important treatment decisions are jointly made in the RG meetings, based on shared decision-making principles (6, 8).

The origins of the RG method lie in the Optimal Treatment (OT) model, which integrates biomedical, psychological, and social strategies in the management of SMI (9, 10). In Sweden, the model was further developed and relabeled as Resource Group Assertive Community Treatment (RACT) (4, 7, 11), in which ACT teams were enriched by resource groups. Research on RACT has focused on effectiveness and found improvements in functioning, well-being, and symptoms for people with psychosis (4, 7). However, the available studies provide little insight into the meaning for and experiences of all those involved when the RG method is implemented. Qualitative contributions to the body of knowledge of the RG method are scarce and have focused on the case managers' point of view (6). Therefore, this study used a qualitative design to explore the perspectives of patients, family, friends, and mental health care professionals when working with the RG method within FACT.

### Study Aims

Based on the limited knowledge in the literature of the meaning of working according to the resource group method, the present study aimed to: (i) identify the general themes of the resource group method from the perspectives of patients, significant others and mental health professionals; and (ii) develop a useful framework for deeper understanding of the resource group method and its outcomes in terms of recovery for people with severe mental illness.

## Methodology

### Context of the Study

This exploratory qualitative study was conducted in the context of a randomized controlled trial (RCT) on the (cost) effectiveness of RGs embedded in FACT for people with SMI in the Netherlands; for a detailed description, see Tjaden et al. (12). To start a RG in FACT, several phases are carried out. In short, patients ask their significant others and mental health professionals to join the RG; this process is referred to as *nominating*. Then, together with a mental health practitioner of the FACT team, patients prepare the first RG meeting by developing a recovery plan to discuss during the meeting, by setting the agenda, and by deciding on the location and chairman (preferably, the patients themselves). Before the meeting, the practitioner invites the nominated RG members for an in-depth conversation about the relationships among the nominee, the patient, and the other RG members, and the role the nominee wants to have in the RG. Follow-up RG meetings are scheduled once every 3 months on average. The composition of the RG is flexible and might change over time depending on patients' goals, wishes, and phase of recovery.

### Design

The study used a multiple case study design, based on the grounded theory (GT) methodology, for the in-depth exploration of processes and developments in eight cases (i.e., eight patients and their RGs). GT is a method that inductively builds an interpretative theory of a social phenomenon, based on qualitative data (13, 14). By following a smaller number of cases for a longer period of time, the researchers aimed to acquire rich data (15, 16) to identify key concepts supporting the theoretical understanding of the impact of the RG method on all those involved.

### Recruitment

After patients completed the baseline assessment of the aforementioned RCT, the first author asked them whether they agreed to participate in the qualitative study. Initially, a purposive sampling strategy was employed among those willing to participate, aiming to include a diversity of patients in terms of sites, diagnosis, gender, and current and past service use. The first author approached seven patients and provided more information about the study; all of them agreed to participate. Two patients dropped out after the first interview and their data was removed; one of them was referred to a different treatment setting, and the other withdrew consent after 1 week. A second round of sampling was conducted after the first few months of data collection and initial analysis. In this round, RGs who could shed light on preliminary categories and concepts were invited. This form of theoretical sampling was made possible because the first author had insight into all the RGs in the RCT. Three additional cases were included in the study after the second round of sampling.

The final sample included eight cases: eight patients and their RGs, comprising 10 informal RG members (i.e., family and friends) and 20 formal RG members (i.e., mental health professionals). Five cases were followed and interviewed by the first author and three by the second author. Cases were followed until within-case saturation occurred [i.e., the moment when new data collection no longer seemed to bring up major new developments in that particular case (17)]; the time period ranged from 6 months to 2 years. In one case, setting up the resource group was repeatedly postponed until after the end of the study; however, we continued to monitor this case and included the data in the study because it offered insight into the impeding factors of the RG method. See Table 1 for a short description of each participant and his/her RG.

**Table 1:** RG composition and short description of the background of each participant^a^.

**Participant**	**RG composition**	**Short description**
Karen	4: Husband, case manager, job coach, and psychologist	Karen is married and lives together with her children and husband. She suffers from severe obsessive cleaning and ruminative thinking. She aims to be a good mother, broaden her world, and be better understood by her husband
John	5: Brother, mother, peer worker, case manager, and job coach	John suffered his first psychotic episode during young adulthood. He lives together with his brother, works as a volunteer, and is doing a vocational study. He aims to travel, have a paid job, and meaningful social relations
Brit	6: Partner, mother, good friend, case manager, peer worker, and psychologist	At the start of her treatment, Brit had not been out of her house for several years. She lives together with her partner. She makes art and writes. She aims to feel free, to be able to go outside without fear, and to develop her (artistic) talents
Martin	6: Mother, stepfather, brother, sister-in-law, case manager, and mentor of volunteer work	Martin suffers from drug addiction and severe depression. He lives with his cat and does volunteer work. He aims to get clean and save money to re-engage in his hobbies
Mandy	None: No RG related activities have taken place during the course of the study	Mandy has had manic periods alternating with severe depressive episodes since she was young. She lives with her son and has changing jobs. She aims to reconnect with herself and to complete a study
Leon	7: Mother, (ex)partner, two friends, case manager, peer worker, and supported living supervisor	Leon experiences frequent dissociative fugue states and has a history of addiction, self-harming, and suicide attempts. During the study period, he moved in with his parents after breaking up with his partner. He aims to have a meaningful job, live independently, and have a satisfying social life
Raoul	4: Mother, brother, case manager, and social worker	Raoul suffered his first psychotic episode during young adulthood, during a period of substance abuse. After living on the street, he now lives in a sheltered housing. He aims to stabilize on medication and to become an peer worker
Martha	3: Case manager, psychologist, and psychiatrist; her partner is invited to participate but does not attend the meetings	Martha has experienced early childhood traumas and suffered from paranoid ideas and severe depression, leading to many hospitalizations. She has two grown children and lives together with her partner. She aims to reconnect with life and become a peer worker

a *Some information (such as profession and living situation) has been modified in order to protect the identity of the participants*.

### Data Collection

Data collection took place between November 2017 and December 2019. All interviews and RG meetings were recorded, transcribed verbatim, and anonymized. The researchers kept memos and field notes throughout the data collection. There was no time limit set for the interviews, the duration ranged from 20 min to 2 h.

In each case, the data collection started with a narrative interview with the patient (18) to get acquainted with his/her life story, most important relationships, wishes for the future, perceived obstacles in life, and expectations of the RG. In the following period, the researchers established a personal connection with the patients built on the co-construction of knowledge and the recognition that the researchers were carrying out research *with* their participants, not *on* them (19–22). To this end, the researchers remained in close contact with the patients throughout the study period by means of telephone calls, app contacts, and low-key, face-to-face visits. The researchers kept notes and memos of these contact moments.

During the RG meetings, the researchers recorded the meeting and took field notes. In between the meetings, the researchers conducted repeated in-depth interviews with the patients to explore their experiences with regard to the RG meetings, the perception of their own goals and aspirations, and their relations with their social environment (23). The interview style was interactive and guided by neutral, open questions; participants were encouraged to discuss topics that they considered relevant.

The last phase of the data collection included in-depth evaluative interviews using an interview topic guide with both patients and their RG members, including informal and formal members. The topic guides were constructed by the researchers after ~1.5 years of data collection and were based on the collected data and the emerging themes and categories; see Appendix 1. See Table 2 for an overview of the data collected per participant.

**Table 2:** Overview of the collected data^b^, sorted per participant.

**Parti-cipant**	**Narrative interview**	**In between interviews**			**RG meeting**	**Evaluative interview**	**Interviews RG members informal**					**Interviews RG members formal**				**Total**
		**Visits**	**Phone**	**Total**			**Partner**	**Mother**	**Brother**	**Friend**	**Total**	**CM**	**PS**	**Other**	**Total**	
Karen	1	3	1	4	2	1					0	3		1	4	12
John	1	4		4	7	1		1	1		2	1			1	16
Brit	1	5	1	6	4	1	1	1		1	3	2	1	2	5	20
Martin	1	2	2	4	1						0	3			3	9
Mandy	1	1	3	4		1					0	1			1	7
Leon	1	4		4	6	1		1		2	3	1	1		2	17
Raoul		3		3	4	1		1	1		2	1		1	2	12
Martha	1	1		1	2	1					0	1		1	2	7
Total	7			30	26	7					10				20	100

b *As we lost contact with one of the participants during the data collection (Martin) we could not conduct the final interview, nor ask his RG members (n = 4) to participate. Moreover, as one of the participants did not start a RG (Mandy) there were no RG meetings to attend and no RG members to interview. Also, one informal RG member (partner Karen) and one formal RG member (peer-worker John) did not respond to our request to interview them despite several attempts. Lastly, one of the participants (Raoul) had invited his mother and case-manager to the narrative interview, and therefore we couldn't follow the topic-guide. We added this interview to the “in-between” interviews*.

### Data Analysis

Data were analyzed using the constant comparative method to identify similarities and differences in the themes emerging from participant experiences. This guided the researchers into more abstract understandings of the themes and the development of more holistic interpretations of the meaning of the RG (13, 14). Data were analyzed chronologically by case, meaning that all data from one case was analyzed in chronological order, after which all data from the next case were analyzed. The MAXQDA software (version 2) for qualitative data analysis was used for coding (24). The consolidated criteria for reporting qualitative research (COREQ) checklist (25) was used to guide the analysis and report.

To conduct the analysis, the researchers first carefully read and re-read the transcripts of all data to familiarize themselves with the material, and they made notes about the content. They developed a global coding frame based on these first impressions and their observations, memos, and meeting notes during data collection. In this coding frame, a distinction was made between *processes, effects*, and *hindering factors*. Subsequently, the researchers jointly coded all 95 transcripts line-by-line, and more detailed codes were generated (“open coding”). When the analysis of the different cases was underway, they compared, combined, and clustered all labels to connect codes and categories and to find potential overarching patterns and themes (“axial coding”) (13, 26). The researchers kept notes of their discussions of the process during the analysis. They continually looked for shared understanding to check the validity of the codes as they were developed, refined, and codified. The benefits of having multiple coders rest in the “content of (coding) disagreements and the insights that discussions can provide to refine coding frames” (p. 1116) (27). Emerging themes were discussed with a wider research team as a validity check.

### Quality Procedures

A number of techniques were incorporated in the study design to increase methodological rigor (27, 28). First, data triangulation was applied by collecting data over the course of 2 years, in various regions of the Netherlands, and by asking different persons to reflect on the same situation. Second, methodological triangulation was applied as we used various methods to gather the data (open interviews, semi-structured interviews, and observations of the RG meetings). In addition, different perspectives were included, covering experiences from patients, significant others, and mental health professionals (25, 27, 29). Third, the internal validity and reliability were enhanced through reflection procedures. The first and second author kept memos of their experiences and discussed these during the study to be aware of their personal frames that shape their interpretations and to be aware of any distortions caused by personal and professional background (29).

## Findings

### Description of Participants

The eight patients ranged in age from 27 to 60 (mean = 37). The duration in mental health care ranged from 5 to 19 years. There was a wide variety of diagnoses, including schizophrenia, addiction, personality disorder, obsessive-compulsive disorder, and mood disorder. The RG of the patients varied in composition and goals; see Table 1.

### Qualitative Results

The analysis showed that in cases in which the specific elements of the RG method were successfully implemented (i.e., the patient nominated members for his/her RG, the RG met regularly, a recovery plan was made, and the agenda of the meetings was set by the patient), five recovery-facilitating processes unfolded that, in turn, provoked effects for individual patients, social interaction, and the provision of care. Three factors emerged from the analysis that might hinder the potential of the RG method. These processes, effects, and hindering factors are reported below and are illustrated by anonymized excerpts from transcripts from patients, significant others, and mental health professionals.

#### Recovery-Facilitating Processes Within Resource Groups

Five recovery-facilitating processes were derived from the analysis: (i) experience of support; (ii) acknowledgment of significant others; (iii) activation; (iv) openness; and (v) integration.

### Experience of Support

The first process concerned the way the support system is mobilized. Seeing their own RG gathered in a room made patients realize they are being loved, acknowledged, valued, and encouraged. That is, the explicit experience of people wanting to be part of the RG conveyed the message to patients that their burden is legit, that one doesn't have to do it all alone, and that there is hope for change. Importantly, the analysis showed that it was the mere presence of the RG members that seemed to provoke this, rather than practical help or actual tasks. “Being there” was the important mechanism, both for the affirmation of lived experiences and psychosocial problems, and for the establishment of a foundation from which change may arise.

John: “What was also nice about it, you know, is that when you join such a resource group, you actually feel that you matter and that you are working on something. Yes, you know, during those meetings you are actually gathered all together. And I think that is also very nice. That you do matter again a bit, so to say, that you don't feel that you are being abandoned, or that no one cares about you. Feeling that there are people around you who are trying to achieve something with you. I think that is also very important. Having that realization, ‘Oh, we're working on something together,’ at least there are people who want to do that with me. […] That you are part of something, so to speak.”

Leon: “In itself, it has certainly been helpful, yes, it certainly helps. Just having all those people in one room. Just the feeling of ‘look at the kind of network I actually have around me.’ To have that in front of you, literally, pictured, and around you, that is very valuable and very supportive.”

### Acknowledgment of the Significant Other

The second process that unfolded in the RG has to do with the firm recognition of the role and position of the significant other in the illness and the recovery journey of the patient. This was initiated during the in-depth conversation prior to the first RG meeting between the significant other and the practitioner from FACT (i.e., one part of starting up an RG includes the practitioner from the FACT team meeting with significant others). Significant others reported that during these interviews they felt that the mental health professionals carefully listened to their side of the story about their loved ones' illness, their experienced burden in daily life, and their personal needs. Moreover, significant others experienced the RG meetings as a stage to share their own experiences, including their concerns, anxieties, and needs. This strengthened their confidence in working together with the mental health professional, who was considered a reliable partner, and softened their attitude toward the patient. The analysis showed that the process of acknowledgment of the significant others was fundamental to establishing readiness for stepping into an active, constructive role as an RG member.

Leon's case manager: “It is good to talk about who can do what. What can we do as mental healthcare providers, what can your network do and, well, what does your partner need to support you in this? What you need is very important, but also what your partner needs in that situation.” Leon: “It is of great importance to see if we can spare her a little.”

Raoul's mother: “Yes on that [living independently], panic just takes over for me.”

Case manager: “Yes, and I did indeed notice that during the personal interview at the beginning. We then concluded that it would be good if we inform you a bit more about that and how we approach it and how it works, so that the steps become more visible. […]”

Social worker: “I absolutely understand your concerns, ma'am. If you have been through all that, I can imagine that you feel very scared and nervous about taking this step again.”

### Activation

The third process is that all involved in an RG were motivated to take on an active role in the recovery process. There were two ways in which the method was found to be activating for the patient: through self-reflection and through commitment toward his/her significant others. The method was also found to be activating for significant others.

#### Activation Through Self-Reflection

Patients related that the RG method motivated them to actively think about their needs, wishes, vulnerabilities, relations, and future perspectives, as they were invited to design their own RG plan, decide which topics would be discussed, and take the lead during the RG meetings. In addition, the presence of their significant others in the meetings motivated the patients to find a way to describe what is wrong, what is hoped for, and what is to be done about it, in a manner that was accessible and understandable by their RG members. The self-reflective processes that emerged from this then led to patients becoming more intrinsically motivated to achieve their own recovery goals and recapture a sense of agency over the topics, actions, and challenges concerning their illness and recovery process. Patients increasingly felt that the recovery process they faced was actually *theirs*, and that they would have to take action themselves if they wanted to see things changed. These self-reflective processes also helped them to distinguish between what changes they were able to make by themselves and what they needed others for.

John: “I really enjoyed making the resource group plan myself. That is a new experience for me because you get to think about things that usually only your practitioner thinks about. And, you have … you actually put things on paper. And yes, that's nice. […] I have the feeling that my brain is slowly starting to work again.”

Brit: “Because God, you get to know yourself well! That is really very bizarre.”

Researcher: “Through the resource group?”

Brit: “Yeah. Yes.”

Researcher: “Do you think that's the most important thing? That you get to know yourself?”

Brit: I think that if you lose yourself or can't ‘read’ yourself … then you get lost. And if you learn to look at yourself from the perspective of ‘what do I actually need to be happy,’ then you can ask for help with that.”

#### Activation Through Commitment

The second way in which the RG method was found to be activating is because patients experienced the RG meetings as periodic evaluation moments in which their recovery goals were shared, evaluated, and further developed with the other RG members. The analysis showed that this committed patients to work on these goals in between the meetings because they felt responsibility toward others, and the presence of others served as an extra motivational impulse.

Brit: “Yes. Plus if you say, ‘well I want to go to the petting zoo,’ you say that in a group of people who all hear you say ‘I want to go to the petting zoo.’ And that then becomes a driving force to indeed try to go to that petting zoo. If it doesn't work, it doesn't work, but you know it gives you something to hold on to. It is difficult, but it is something that, for me, works very well.”

Raoul's brother: “[…] And I can imagine that if you do all this by yourself that you are more inclined to think ‘I can postpone it for a while.’ But now we are all together, and I think that gives him direction and focus when working on his vision for the future. I think it activates him—that might be a better word—it activates him and also us.”

John: “The risk for me is mainly that I feel that I am completely free again, and that I continue to live as if nothing happened. And I think the RG is really important in this. Because you have that responsibility to each other, I have the responsibility to you all, and I really can't let it go wrong.”

#### Activation of Significant Others

Finally, the RG method also activated significant others in two ways. First, the setting of the RG and the encouragement by professionals to explore the interactivity of encountered problems meant that the closest significant others reflected on their own role in these problems. They gained new knowledge and improved and adjusted their behavior and coping skills. Importantly, this did not always imply an increase in the significant other's active behaviors. In certain relations, it meant creating more distance or establishing firmer boundaries. Activation of the significant other is thus to be understood as being activated to reflect and learn about one's own role in encountered problems.

Researcher: “And would he involve you in certain goals? Raoul's mother: “He doesn't do that quite so quickly, and I understand that. I may have to intervene less rather than more with his issues; that would be very nice for him, I think.”

Secondly, significant others outside the circle of the main caregivers (e.g., friends) were invited to become part of the support system as well. They were present during the meetings and involved with the discussion about how to achieve the goals. Moreover, they were encouraged to share their opinions, feedback, and possible concerns. This made them active collaboration partners in the patient's process rather than passive bystanders.

Brit's friend: “I'm happy to be able to help. She asked me to go biking together once a week, and after that I will stick around for a while because it is just fun [...]. And I also notice that biking is becoming easier for her, because she likes to do it with me.”

### Openness

The fourth process that unfolded in the context of the RG is a breakthrough of mutual communication patterns within the informal support system. The setting of an RG meeting set the stage for honesty, mutual disclosure, and candid discussions within the safety of the patients' support system. Although many patients described feeling tense to be open and talk about their vulnerabilities, the setting served as an invitation to all RG members to jointly explore a way to open up and address difficult events or feelings in their lives. This openness had to do with both the patient's recovery process and the perspective of all RG members concerning struggles from the past and the role they could play in the recovery process. This way, expectations and responsibilities were discussed, adjusted, and approved; and patients had the experience that sharing difficulties does not indicate a sign of weakness but is part of the person, who is liked and valued by others. The openness in the RG meetings about both the good and the bad internalized the message that they can be ill and well at the same time because it is part of their total self. Importantly, the analysis suggested that the previously described processes of acknowledgment and activation were both essential prerequisites for the process of openness to emerge as these induced readiness to become equal partners in the open interaction and take on a meaningful role in the dialogical process.

Raoul: “I think it is nice to have a set time for everyone to be honest and open so the difficult things don't interfere in the meantime. And I found out that my family is not good at discussing these things directly with each other. Now we all have a say in those meetings; yes, that's good, and I like it.”

Martin's brother: “I think it is great that you're telling us what you want [some distance from the family] but at the same time I think, ‘Well, that is easily said,’ because for me, I find it very difficult. And why do I find that difficult, because, and now I am going to say something very personal, but you have had suicidal tendencies. And for me it is really scary to leave you alone for a long time. […] I think it's scary if I haven't spoken to you in a week. When I am at your door, I think, ‘Maybe he is lying there on the floor and I have lost my brother [tears in his voice].’ Do you understand?”

Raoul's brother: “So the vulnerability that he shows now, that is something he never dared to or could have shown before. So yes, absolutely, that's the biggest difference I've seen. The meetings really trigger that, or maybe it was already there, and give the meetings a stage for all of us to be a bit more vulnerable, I don't know.”

### Integration

The final process evoked by the RG method is that a more unified support system around the patient. Characteristic of having a severe mental illness is facing difficulties in multiple domains in life. Gathering the people that belong to these different domains facilitated a better representation of the different parts of a recovery journey and encouraged the search for one's integrated narrative. Patients felt that all RG members obtained a new, improved understanding of their situation when the significant others and involved professionals met on a regular basis because it was felt to be a more complete representation of who they are. Moreover, it allowed RG members to place different parts in the context of the bigger picture and facilitated integration of both healthy and sick parts of their recovery journey toward a coherent storyline within a recovery process. In this way, the recovery journey as a whole was affirmed.

Raoul's social worker: “Well, now it's more of a system, it's not just him, but it's all of his system around him. And that makes you feel more. Yes, how do you say that. as if you now know more about his life. Normally, it was something Raoul said, and I never knew the other side, and now I get to see that his mother has a completely different view on things, which is also partially true. So now, the story has become more complete.”

Brit: “I think it is very important that as a patient you don't always feel like a patient, that you are really seen as a person and that they also try to see what her character is and what fits in there. […] And now we are really looking at ‘who is Brit, what does actually work for her.”’

Not only did the RG method ensure better integration at the level of the personal story, it also served as a platform for better integration of the professional disciplines involved. The regular meetings provided a stage for the adjustment of and more comprehensive communication about treatment and care aspects within the context of the patient's narrative and his/her social environment and everyday life.

Martha's case manager: “I think if the psychologist weren't part of the RG, Martha would be on higher levels of medication than she is now. Martha and the psychiatrist now dare to try to lower her medication level. I believe that the encouragement and confidence of the psychologist have been decisive in reducing the level of medication. That's why I like that we are gathering together.”

#### Effects of the Resource Group Method: Where Did the Emerging Recovery-Facilitating Processes Lead?

The analysis produced three themes that represent effects of the RG method: (i) arousing curiosity about the world beyond illness in patients; (ii) steps toward reciprocity and equality in their social relations; and (iii) a framework for recovery-oriented mental health care. It is important to keep in mind when interpreting these results that these effects cannot be attributed unilaterally to the RG method itself. The analysis showed that other factors, such as the backgrounds, experiences, and characters of those involved, and the patient's readiness for change also play an important role in achieving these successes.

### Arousing Curiosity in Patients

Patients with a well-functioning RG seemed to develop, after a while, an increased interest in participating in the world beyond mental health care. Although the RG method did not lead to recovery in a specific domain, an enhanced overall curiosity was identified in patients who worked with an RG. The processes initiated by the RG method seemed to establish a feeling of being worth it to participate and to enhance self-confidence, which, in turn, aroused a curiosity to (re)discover one's place in the societal world. As such, participants related that the RG awakened them, set them in motion, and motivated them to reconsider their situation and themselves.

Brit's case manager: “I can see that she has grown a lot in realizing that she actually is someone and that she is allowed to be. That she is allowed to be part of society even if for now only in a limited way. But that she realizes that the world is bigger than just her apartment and the internet.”

Leon: “That space has grown in my head.”

Researcher: “And why is that?”

Leon: “It feels as if I have woken up a bit. I now wonder what is going on in the outside world. And I am discovering little by little what part. yes, what is in it for me.”

Researcher: “And has the RG played a part in that?”

Leon: “Yes, yes, I do think so. It just triggered me to do things, and I've found that when I discuss things with other people that the world kind of becomes a bit bigger.”

### Steps Toward Reciprocity and Equality in Mutual Social Relations

In most of the RGs that were studied, a shift took place over time from a relationship of dependence to a more reciprocal interaction between patients and their relatives, in which not only the patient but also the relatives could have and show their vulnerabilities. It was observed that the processes initiated by the RG method enhanced the relatives' trust and released them from the task to be constantly alert. This, in turn, decreased tension and stress in their contact with the patient and created space wherein a more equal relationship could evolve. The RG method thus seems to have the potential to make difficulties and vulnerabilities a human feature: something that is shared and that deepens mutual relationships.

Leon's peer worker: “[…] if you are open to your network and your network is open to you, then, what I just told you about my own friends, then the relationship deepens. For you, but also for the other person. All people have the need for deep, meaningful friendships. You create these together in this way. And I really mean that.”

Raoul: “I'm finally out of that deep hole I was in, so they can count on me again. That feels nice indeed, that it is more equal now. It's not just them helping me, but also me helping them. So it's not one-way anymore.”

The analysis showed the first steps of a (re)building of mutual relationships *beyond* the illness. In many of the RGs, the processes described above created space to jointly explore how to relate to the other in a relationship that was no longer defined by the illness and in which people started doing fun activities together again. Often this was preceded by RG members mutually reinventing their shared interest and a joint search of how they could shape those together.

John's brother: “But when I see myself now, compared to a year ago. I feel connected with him again; we interact more normally and there is much less stress. […] And then come moments when you can do something fun together again. We went to the cinema together last week.”

### Framework for Recovery-Oriented Mental Health Care

The most notable outcome in terms of mental health provision is that the RG method gave mental health professionals a framework in which to work according to the recovery-oriented principles of agency of the patient and involvement of significant others. The structure of the RG ensured a shift toward the context of patients' everyday lives. Explicitly inviting significant others into treatment and care implied that the most important people that accompanied the individual in his/her recovery journey were no longer mostly professionals whose presence was warranted by the person's problems. Instead, the presence of relatives and friends emphasized the uniqueness and multiple facets of one's identity, life stories, and competencies. Mental health care was sensitized to adapt to the uniqueness of the recovery journey and to see an individual within his/her personal context. As a result, a true connection could develop between patients and the professional, comprising curiosity for a person as an individual and sincere attention to what works for them. Although professionals related that these recovery principles were also considered important in their routine services, the method anchored them as the fundamental points of departure of their work.

John: “I think that by means of such an RG you get to know someone much better, you know, multiple sides of someone. You can clearly see that every person is different. If you apply the RG to someone else, you will probably get very different results.”

Martha's case manager: “I do think that it contributes to an improved quality of treatment. As I said, you consider those close to the patient, and that is so important, and you really take time, you consistently focus on truly understanding and acknowledging the person and his/her wishes for development. Organizing the meetings, gathering together, and the cooperation actually force you to do so.”

#### Hindering Factors in Establishing an RG

The analysis revealed three factors that interfered with establishing an RG that would serve as a safe basis for unfolding the recovery-facilitating processes and effects as described above: (i) embedding and implementation issues; (ii) predominant network; and (iii) tensions inherent to the RG setting. These reflect domains of attention when working according to the RG method for people with SMI, especially in the initial phases.

### Embedding and Implementation

The analysis showed that mental health professionals experienced an increased workload when incorporating the RG method into the routine practice of managing patient symptoms and basic needs (such as housing, hygiene, and medication). Mental health professionals reported that the method demanded extra time, particularly in the initial phase, to thoroughly prepare the RG meetings with the patient and to establish a good working relationship with significant others. Although they felt that it contributed to what they perceived as good mental health care, and they considered the extra time to be a valuable investment, they were hindered by high caseloads, recurrent staff turnover, and organizational issues, such as reorganization and lack of management support.

Researcher: “And do you plan to expand this in your work?”

John's case manager: “I would like that for the future, but I actually feel overloaded at the moment; it is not feasible.”

Researcher: “Time wise?”

John's case manager: “Yes, I just don't have the time for that. [...]. So things like that … yes well, that it's just not possible. It is frustrating, though. I mean, there are more things you don't get around to. Because in essence, the concept is simply beautiful.”

Mandy's case manager: “In the first instance, I have to take my own share of the blame; I actually have not had room for this [implementation of the RG method]. I know from my own experience, because I've done it before on another team, that when you start, you really need to have space and time, which I just haven't had in the past period.”

### Predominant Network

The analysis revealed that several forms of complexity within the support system could interfere with establishing a well-functioning RG. The first is significant others that were too agitated, anxious, judgmental, or distressed during the RG meeting. The RG meeting was then no longer about the patient's issues and recovery, but was interfered with those of the significant others. Moreover, tension between the informal RG members—including feelings such as blame, disappointment, and disagreement—and unwillingness of the informal RG members were both found to be complicating factors. The data showed that thorough preparation and collaboration with significant others was fundamental to decreasing their emotions and frustrations and obtaining readiness to constructively contribute to the patient's recovery process. When overlooked, the RG method could aggravate the existing complexities, which stood in the way of an empowering and safe environment in which patients could work on their recovery process.

Leon's friend: “Yes, sometimes I had to bite my tongue. My frustrations … Yes, I did not really consider it to be the place to express them, but I sometimes found it difficult to deal especially with his mother. [...] And then I feel like, I should not mention it here because it is already difficult for Leon and of course you do not want to have an argument about him. But that sometimes makes you go there with a bad taste in your mouth, yes. So I sat there … well yes, more negative.”

### Tensions Inherent to the RG Setting

Finally, the analysis showed that the setting of the RG could be stressful for patients and evoke feelings of vulnerability, insecurity, and weakness. This was especially the case when psychiatric or psychological symptoms and associated problems, such as suspicion, anxiety, low concentration, changes in medication, side effects, and abrupt alterations in goals, wishes, and motivation were not sufficiently recognized and acted upon. As a result, patients were placed in a position they were not able to live up to, which compromised the patient's agency and evoked feelings of blame, disappointment, and misunderstanding in significant others. This complicated the establishment of a well-functioning RG.

*Excerpt from field notes about Karen: In my experience, her feelings of inferiority are very much in the way of a healthy and fertile RG trajectory. She is not (yet) at all on the track of experiencing the RG as a group of people who can support her in her process. Rather, she feels subjected to the RG structure and everything that goes with it*.

Leon: “Yes, the exam feeling. Just like, ‘did I pass the past period or not?’ I think that's a little how it feels. Yes, and every time I felt like I had taken a step back, it felt like I had to justify why I ‘failed’.”

## Discussion

In this paper, we aimed to gain a deeper understanding of the RG method when integrated into FACT. Based on the data obtained from observations of RG meetings and interviews with patients, their significant others, and mental health professionals, our findings indicate that a well-functioning RG sets the stage for five processes to unfold: experience of support, acknowledgment of significant others, activation, openness, and integration. These processes, in turn, facilitate patients' entrance into what can best be described as a “pre-phase” of recovery: they develop an arousing curiosity about the world beyond illness and, together with their significant others, rediscover forms of reciprocity and equality in their social relations. Of particular relevance is the finding that the method emphasizes and reinforces the uniqueness of each person's context and recovery process, thereby providing a framework for the provision of recovery-oriented care. However, it is not self-evident that a well-functioning RG will be established. There are at least three hindering factors that should be addressed and overcome: implementation issues, a predominant network, and tensions inherent in the RG setting.

Overall, the study showed that working according to the RG method anchors the view of mental health and recovery as a contextual and relational phenomenon. This cultivates a shift of treatment and care toward the context of patients' social environment and everyday life. As a result, the person's recovery work takes place in relation to the other people in his or her surroundings. The recovery path, including both recovery and relapse, inherently becomes a social process in which all RG members are important and equal partners whose needs are acknowledged and integrated within the journey. Importantly, conceptualizing recovery as a social process doesn't imply that the patient's recovery path is necessarily related to an increase in collective or social experiences. For some, working on their recovery meant disconnecting from certain relationships, establishing firmer boundaries, and growth in autonomy and self-determination. Nevertheless, these alterations are all located in the context of community, family, and other relationships [see also (30, 31)]. In this way, it was not only the individual patients going through a recovery process but also their social network. The RG method offered an opportunity to align these co-existing but interdependent processes and to construct a mutual story, in order to create space for long-lasting changes within the environment of everyday life.

In much of the literature, recovery is perceived as a process that takes place within and by the individual and in which autonomy, responsibility, and self-determination are essential elements. In what has become a classic definition, Anthony (32) described recovery as “a deeply personal, unique process of changing one's attitudes, values, feelings, goals and/or roles. It is a way of living a satisfying, hopeful, and contributing life even within the limitations caused by illness. Recovery involves the development of new meaning and purpose in life as one grows beyond the catastrophic effects of mental illness” (p. 4). Here, the individual essence of recovery of SMI is emphasized. Based on our study, one should assume a deeply *social* process rather than a deeply *personal* process, in which concepts such as autonomy, responsibility, and self-determination become meaningful in the context of relationships; consequently, they cannot be regarded as isolated goals of the recovery process. As Schön et al. (33) argued, “It is through social relationships that the individual is able to redefine themselves as a person (as opposed to a patient)” (p. 345). In other words, the social world is the medium through which transformation becomes possible. Importantly, this transformation concerns not only the patient; the social network is also subject to change in order to facilitate, acknowledge, and live with the transformation.

Our findings are in line with the increased recognition of the importance of including the context of community, family, and other relationships in understanding, analyzing, and responding to mental health difficulties and recovery (30, 31, 34). Family members' emotions, behaviors, and attitudes toward mental illness are among the strongest predictors of both relapse and recovery for people with an SMI (35), and the social and contextual nature of recovery has been underlined (31, 36, 37). As such, this highlights an essential task of mental health care: to facilitate social environments within which recovery is enabled (38). Our study suggests different processes that are important in creating these enabling environments in order to develop equal partnerships between mental health service providers, service users, and significant others.

### Limitations

The findings of the study should be viewed in light of some limitations. First, the uniqueness of the recovery journeys of the participants and the small sample size limits the generalizability of our findings to a wide population of people with SMI. Although we were able to identify common and shared processes and effects by participants and have thus reached a certain level of theoretical saturation, the findings of this study are rooted in time, place, and person. Hence, future studies should investigate the role of specific characteristics, such as social network size, and also different clinical diagnoses for further application of the RG method. Second, in qualitative research, the researcher is a central figure that influences, if not actively constructs, the collection, selection, and interpretation of data (39). In addition, we were unfortunately not able to conduct a member-check meeting as we had intended, due to the COVID-19 crisis. Although we embedded a number of precautions in our study design to reduce the risk of biased interpretations, it cannot be ruled out that the data interpretation and meaning construction are contingent on the subjectivity of the researchers.

Third, as the first and second author followed patients and their significant others for a longer period of time, they became trusted partners in the development of the participants (both patients and their significant others). This was one of the main strengths of the study because we could develop confidential relationships with the participants, which enabled the disclosure of deeply personal information and vulnerabilities. At the same time, the sincere attention and interest for the participants and the repeated visits might have had a therapeutic influence and contributed to an enhanced sense of self and feelings of social connectedness. In addition, the researchers repeatedly asked to evaluate and reflect on the RG method and its influence on the recovery journey, which may have evoked reflections and attributions to the method that participants otherwise would not have interpreted that way.

### Implications for Clinical Practice

#### The Role of the Mental Health Professional

The five processes identified in the study require the redefinition of roles, responsibilities, and mutual relationships in the context of care provision. That is, the dynamic between professionals, patients, and significant others is reshaped to “doing *with*, rather than doing *to* and doing *for*” [(40), p. 41]. This demands a shift in attitude of the mental health professional when compared to a more individualistic, focused treatment. It requires that professionals decenter their professional expertise and instead take on the role of monitoring the processes within the RG in order to establish the conditions that enable the patient to take the lead. Although this is a very active role, this activity does not concern determining or controlling the outcomes of the process. Instead, it includes helping to reflect on decisions, recognize vulnerabilities, and incorporate different perspectives. The challenge for the mental health professional here is the simultaneity of their work at the individual patient level and at the RG level. Above all, building on a safe environment for facilitating the patient's recovery process should be preserved as the main aim of the RG meetings, and elements that are affecting this warrant thorough preparation and attention.

#### Organizational Issues

In addition, our study suggests that the RG method needs to be embraced by the workplace and firmly included in work routines in order to be implemented, as with other family-oriented practices (41). When the workplace does not encourage the RG-related activities, providing a training program for an individual professional is not sufficient. Thus, for sustainable implementation, there is a need to develop clear practical guidelines to obtain insight on how to integrate elements of the RG method into outreaching services as usual, including the related organizational challenges.

## Data Availability Statement

The datasets generated for this study are available upon request to the corresponding author.

## Ethics Statement

The studies involving human participants were reviewed and approved by The Medical Ethical Committee of VU Medical Centre (IDS: 2017.316). The patients/participants provided their written informed consent to participate in this study. Written informed consent was obtained from the individual(s) for the publication of any potentially identifiable images or data included in this article.

## Author Contributions

CT and JB were responsible for data collection, performed the interviews, analysis, and wrote the first draft of the paper. CM and HK were involved in the several rounds of analyses and provided comments on drafts of the paper. All authors contributed to the study design and concept development.

## Conflict of Interest

The authors declare that the research was conducted in the absence of any commercial or financial relationships that could be construed as a potential conflict of interest.
