# Supplementary material for: Embracing the Social Nature of Recovery: A Qualitative Study on the Resource Group Method for People With Severe Mental Illness
Source: Front Psychiatry. 2020 Nov 24;11:574256. doi: 10.3389/fpsyt.2020.574256 (PMC7732607; doi:10.3389/fpsyt.2020.574256)
Supplement: Supplementary file 1 [file Data_Sheet_1.docx]

# Appendix 1: Topic lists interviews

## 1.1: Topic list narrative interview patient

Part 1: Present

- How does your life look like now, what do you do, daily activities, living situation, etc

Part 2: Course of life

- What have been important events in your life
- Drawing of the life line
- Important relations throughout your life

Part 3: Future

- Future in 5 years: how does that look like, what has changed, what has remained the same
- What do you need to find meaning in life

Part 4: Resourcegroup

- Description of the composition and why
- How are the relations with and between members
- Expectations/hopes/themes to be discussed in the RG

## 1.2 Topic list final interview patient

Part 1: Reflections on the RG

- How would you describe a resource group?
- We started the RG (…) years / months ago, first the interviews with your significant others, then the planning of the meeting, and the meetings themselves. How did you experience the starting phase of the group?
  - Meeting structure
  - Gathering together
  - Personal relation with your casemanager
- Most important developments in the RG
- RG versus previous care: most important change?
- What you do need to start an RG?

Part 2: Influence of the RG on your recovery process

- What does having an RG offer you? What has been its influence?
  - Have you seen a different side of yourself?
  - Has something been started? (social, personal, clinical recovery process). If so, what / how / why?
  - More control over treatment? And life?
- Could you describe your new struggles, new conflicts, new challenges
- RG and moments of crisis/you are feeling severely bad
- Change in interest in the social world?

Part 3: Influence RG on relationship with the people of your group

- Course of the relationship with the people in your group / influence RG on it
  - Normalizing the relationship
  - Or professionalize (stigmatization? Control?)
  - Feelings of dependence

Part 4: Good mental health care

The aim of the research is to improve care. What do you think about the most important steps that still have to be taken?

## 1.3 Topic list interview significant others informal support system

Part 1: Reflections on the RG

- How would you describe a resource group?
- We started the RG (…) years / months ago, first the interviews with your significant others, then the planning of the meeting, and the meetings themselves. How did you experience the group?
- Most important developments in the RG
- RG versus previous care: most important change?
- Good sides of it; improvements?
- Most important role within the RG? (Do you like that/can you handle it?)
- What is needed to start an RG?

Part 2: Influence of the RG on the recovery process

- What does having an RG do with your significant other? What has been its influence?
  - Have you seen a different side of him/her?
  - Has something been started? (social, personal, clinical recovery process). If so, what / how/why?
  - More control over treatment? And life?
- RG and moments of crisis/your significant other was feeling severely bad

Part 3: Influence RG on relationships

- Course of the relationship with patient/influence RG on it
  - Normalizing the relationship
  - Or professionalize (stigmatization? Control?)
  - Feelings of dependence

Part 4: Influence RG on your personal well-being

- Well-being/burden of being a caretaker
  - Experience of caretaking and supporting, can you handle it, is there enough space for yourself?
- Acceptation
  - Feelings about psychological problems of your significant other

Part 5: Good mental health care

The aim of the research is to improve care. What do you think about the most important steps that still have to be taken?

## 1.4 Topic list interview mental health professional

Part 1: Reflections on the method

- We started the RG (…) years / months ago, first the interviews with the significant others, then the planning of the meeting, and the meetings themselves. Experiences with working according to the method.
- How would you describe a resource group/definition
- Most important developments in the RG
- RG versus previous care: most important changes in your work
- Good sides of it; improvements
- Most important role within the RG (Do you like that/can you handle it?)
- Hindering and facilitating factors
- RG and your team (implementation)
- Is the RG needed within FACT?

Part 2: Influence of the RG on the recovery process

- What does having an RG do with your patient? What has been its influence?
  - Have you seen a different side of him/her?
  - Has something been started? (social, personal, clinical recovery process). If so, what / how/why?
  - More control over treatment? And life?
- RG and moments of crisis/your significant other was feeling severely bad

Part 3: Influence RG on relationships

- Course of the relationship with patient/influence RG on it
  - Therapeutic relationship
  - Relationship patient and his/her sign other

Part 4: Influence RG on your profession

- - Changes in your work: better/less
  - Additions of the RG in your work

Part 5: Good mental health care

- Future of the RG
- The aim of the research is to improve care. What do you think about the most important steps that still have to be taken?
